# Genetic legacy of cultures indigenous to the Northeast Asian coast in mitochondrial genomes of nearly extinct maritime tribes

**DOI:** 10.1186/s12862-020-01652-1

**Published:** 2020-07-13

**Authors:** Stanislav V. Dryomov, Elena B. Starikovskaya, Azhar M. Nazhmidenova, Igor V. Morozov, Rem I. Sukernik

**Affiliations:** 1 Laboratory of Human Molecular Genetics, Institute of Molecular and Cellular Biology, SBRAS, Novosibirsk, Russian Federation; 2 Institute of Biological Chemistry and Fundamental Medicine, SBRAS, Novosibirsk, Russian Federation; 3 Novosibirsk State University, Novosibirsk, Russian Federation

**Keywords:** Mitochondrial genomes, mtDNA lineages, Russian Far East, Native people, Phylogeography

## Abstract

**Background:**

We have described the diversity of complete mtDNA sequences from ‘relic’ groups of the Russian Far East, primarily the Nivkhi (who speak a language isolate with no clear relatedness to any others) and Oroki of Sakhalin, as well as the sedentary Koryak from Kamchatka and the Udegey of Primorye. Previous studies have shown that most of their traditional territory was dramatically reshaped by the expansion of Tungusic-speaking groups.

**Results:**

Overall, 285 complete mitochondrial sequences were selected for phylogenetic analyses of published, revised and new mitogenomes. To highlight the likely role of Neolithic expansions in shaping the phylogeographical landscape of the Russian Far East, we focus on the major East Eurasian maternal lineages (Y1a, G1b, D4m2, D4e5, M7a2, and N9b) that are restricted to the coastal area. To obtain more insight into autochthonous populations, we removed from the phylogeographic analysis the G2a, G3a2, M8a1, M9a1, and C4b1 lineages, also found within our samples, likely resulting from admixture between the expanding proto-Tungus and the indigenous Paleoasiatic groups with whom they assimilated. Phylogenetic analysis reveals that unlike the relatively diverse lineage spectrum observed in the Amur estuary and northwestern Sakhalin, the present-day subpopulation on the northeastern coast of the island is relatively homogenous: a sole Y1a sublineage, conspicuous for its nodal mutation at m.16189 T > C!, includes different haplotypes. Sharing of the Y1a-m.16189 T > C! sublineages and haplotypes among the Nivkhi, Ulchi and sedentary Koryak is also evident. Aside from Y1a, the entire tree approach expands our understanding of the evolutionary history of haplogroups G1, D4m, N9b, and M7a2. Specifically, we identified the novel haplogroup N9b1 in Primorye, which implies a link between a component of the Udegey ancestry and the Hokkaido Jomon.

**Conclusions:**

Through a comprehensive dataset of mitochondrial genomes retained in autochthonous populations along the coast between Primorye and the Bering Strait, we considerably extended the sequence diversity of these populations to provide new features based on the number and timing of founding lineages. We emphasize the value of integrating genealogical information with genetic data for reconstructing the population history of indigenous groups dramatically impacted by twentieth century resettlement and social upheavals.

## Background

“Northeastern Paleoasiatic people” refers to a heterogeneous set of populations in the Russian Far East and eastern Siberia, including the Nivkhi in the Lower Amur region and Sakhalin, the Yukaghir, and Chukotko-Kamchatkan peoples. The Chukotko-Kamchatkan peoples are thought by many investigators to have had a special relationship with the ancient inhabitants of a vast portion of Northeast Asia before this territory was dramatically reshaped by the expansion of Tungusic-speaking groups [1–3]. The contours of Northeastern Paleoasiatic ethnogenesis become archeologically visible at the onset of the Neolithic era (beginning in the late Pleistocene in the Russian Far East but regionally varying, with early Neolithic onset in Sakhalin at ~ 7000–5000 BC) [4]. In this period, the predominant tundra belt between the Arctic Ocean and Anadyr River and the taiga zone between the Anadyr and Koryak Mountains were characterized by nomadic hunters mainly pursuing wild reindeer, supplemented by some inland fishing and plant gathering. Coastal areas extending from Chukotka remained largely uninhabited until the late Neolithic [3]. The core of this model is represented by diverse sedentary Koryak groups occupying the northern coasts of the Okhotsk Sea. The Koryak prehistory reflects a long stage of fishing and hunting cultures of the Neolithic and post-Neolithic periods, followed by Tokarev (seventh c. BC to second c. AD) and subsequent Old Koryak cultures (formed in the early 1st millennium AD) that introduced specialized marine animal hunting. The Old Koryak cultures extended along the western coasts of the Sea of Okhotsk to reach Sakhalin Island and played a key role in the formation of “Okhotsk culture”, a contested collective designation for forager-fisher cultures with strong marine orientations across Sakhalin, Hokkaido, and the Kurile Islands in the mid-1st to early-2nd centuries AD [5–7].

### The Nivkhi

The traditional area of Nivkhi inhabitance consists of two main territorial subdivisions – the mainland subgroup dispersed up to 100 km in the lower course of the Amur River area and a coastal subgroup living mainly along the northwestern and northeastern coasts of Sakhalin Island. In traditional times, the Nivkhi were sea mammal hunters of the Lower Amur/Southern Okhotsk region and numbered in the several thousands. Despite territorial and political claims to Sakhalin from the Mongol and Manchu Empires, the Nivkhi remained the numerically predominant aboriginal people until the modern colonial period, when influxes of Russians from the north and Japanese from the south reduced them to minority status.

The first Russians to write about the Nivkhi in the mid-seventeenth century called them “Gilyak” (a Tungus exonym), by which they would be referred until 1930 [8]. Records from the second half of the nineteenth century show a decline in the population size, dropping to 3270 [9]. In 2002, the Nivkhi community increased to 4902, with roughly half living on Sakhalin and half on the mainland. The Nivkhi may never have been widespread on the mainland beyond the coastal belt and Amur estuary, in contrast to Sakhalin Island, which was probably entirely inhabited by ancestors of the Nivkhi before the colonization of its southern regions by the Ainu from Hokkaido during the eleventh century. The Nivkhi language is a true isolate, a linguistic lineage outside the world’s major language families, with no demonstrable genealogical relation to either neighboring or geographically distant languages [10]. Although pre-Holocene archeology is documented from central and southern Sakhalin, the earliest archeological dates for Northern Sakhalin are Neolithic [11].

### The Koryak

Prior to the Tungus’s appearance in the 15th and 16th centuries, the Sea of Okhotsk was inhabited by the coastal Koryak as far as the Nivkhi ethnic border on the Uda River. The ethnic composition of the coastal Koryak comprised dialectally and culturally diverse groups of sedentary river fishers and (to a lesser extent) sea hunters, who gradually assimilated and converged into a broader generic group [12–17]. As a result of the northeastern spread of the Tungusic people, a large segment of the Koryak population was assimilated, and the coastal Koryak territory became greatly reduced, effectively ending the Old Koryak culture there [12]. By the turn of the twentieth century, Reindeer Koryak, close enough to Chukchi, inhabited the forest tundra zone of northwestern Kamchatka and the Penzhina River basin and the northeastern part of the Kamchatka mainland [12, 14].

### The Udegey

The ethnonym Ude (Udi, Udiha) was originally mentioned during the fifth century in reference to a coastal tribe on the Sea of Japan. The early history of the Udegey is thought to be similar to that of other members of the Amur complex (closely related Tungusic-speaking sedentary populations mostly inhabiting the Lower Amur region), as they are presumed descendants of both fishing and hunting groups who inhabited the area since Neolithic times. All the variants of basic Udegey cultures, including one primarily based on sedentary river fishing among lowland groups, one based on forest hunting among mountain groups, and another based on sea mammal hunting among coastal groups, point to a cultural origin common to other Amur groups such as the Ulchi but separate from their Tungus and Manchu neighbors [18]. Hence, the Udegey lineage structures are very complex, with most lineage segments ultimately claiming descent from lineages of other ethnic groups. One reason for the interlineage and interethnic fusion is, as among all other Amur groups, the importance of the exogamous alliances among lineages, which frequently cut across ethnolinguistic boundaries [3]. At the present time, the Udegey number no more than 1000 people. The Udegey groups’ original language belongs to the Tungusic family. However, most of them have already been assimilated into the majority of surrounding Russian speakers.

### The Oroki (Ulta)

The ancestors of today’s Ulta were a group of Tungus-influenced Ulchi who migrated to central Sakhalin with their reindeer during approximately the sixteenth century. Present-day Oroki are among the smallest and most demographically precarious native tribes in Siberia. In 2002, their population size was ~ 200 [8, 19]. They speak a language belonging to the southern subdivision of the Tungusic language family. Notably, many of the Oroki (as well as Nivkhi villages located in the Amur estuary) were susceptible to the impacts of flooding and sometimes completely wiped out, as in the Amur flood of 1968. Hence, we used the birthplace of their maternal grandmothers from abandoned settlements as the location identifier for each complete mitochondrial DNA (mtDNA) sequence, thereby providing information about its location prior to the birth year of the sample donor.

**Fig. 1:**
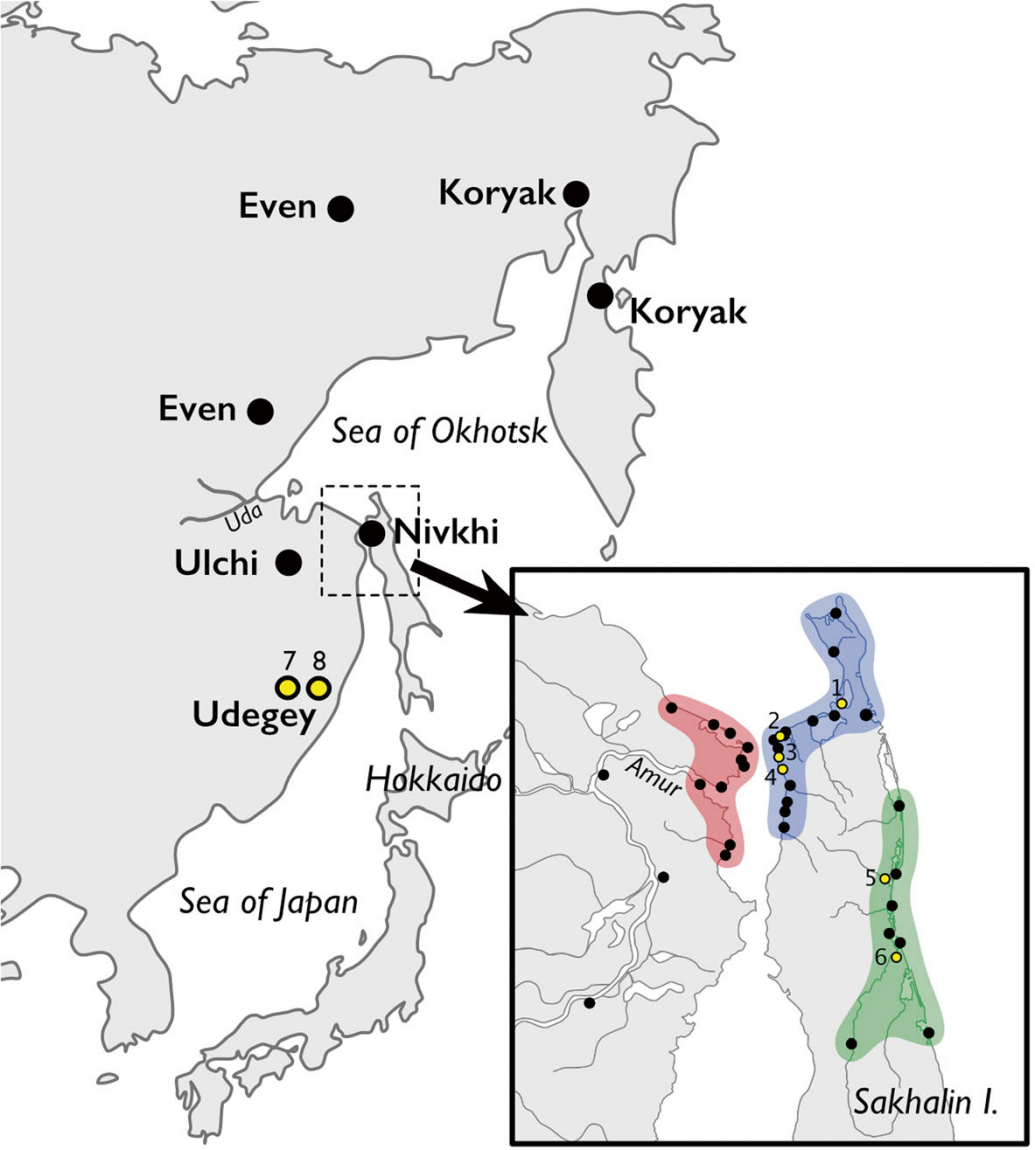
Map of the Russian Far East and adjacent part of the North Pacific, showing mtDNA sampling locations. The enclosed inlet shows how birthplaces of the maternal grandmothers of study participants relate to the documented geographic distribution of distinct Nivkhi dialects. Yellow circles mark the locations of sampling expeditions: 1-Nekrasovka, 2-Rybnovsk, 3-Rybnoye, 4-Lupolovo, 5-Nogliki, 6-Val, 7-Gvasiugi, and 8-Agzu. Black dots denote locations of the abandoned settlements, with the birthplaces of donors highlighted in red on the mainland, blue on northwestern Sakhalin, and green on the eastern coast of the island. Additional information on sampling collection was reported in previous works [20–22]. Many Nivkhi settlements, mostly located in the Amur estuary, were susceptible to flooding and sometimes completely wiped out, as in the Amur flood of 1968. In the process of twentieth century centralization, the Nivkhi and Oroki were forcibly displaced from their more widely distributed settlements into more consolidated locations [8]. This map was made using Affinity Designer version 1.7.3 (https://affinity.serif.com/designer). Data were obtained from OpenStreetMap (https://www.openstreetmap.org/)

To fill the phylogeographic gap between Kamchatka and Sakhalin toward the coast of the Sea of Japan, 56 mtDNA samples from the northwestern Sakhalin and the Amur estuary are revised and synthesized with 52 new blood samples (46 Nivkhi and 6 Oroki) drawn in fall 2016 in the Nogliki and Val settlements, Nogliki District, Sakhalin Region, Russian Federation (Fig. 1). Samples from 93 Nivkhi and Oroki were subjected to complete sequencing: 52 from the current study and 41 previously published. In addition, 27 samples of sedentary Koryak were chosen from a much larger set of previously collected Koryak samples not yet examined at the entire mtDNA genome level [23] and were completely sequenced. Finally, mtDNA from 46 Udegey from our earlier studies in the Sikhote-Alin Mountains in the lower and southern portions of the Amur Basin [20–22, 24] were revised and supplemented by 13 new samples drawn in March 2018 from the village of Agzu, Terney District, Primorsky Krai (Fig. 1).

## Results

The total data set (*n* = 285) was assigned mainly to lineages Y1a, G1b, D4m, D4e5, N9b, and M7a2 (Additional file 1: Table S1). To focus our phylogenetic analyses, we removed G2a, G3a2, M8a1, M9a1, and C4b1, representing likely admixture among proto-Tungus peoples expanding from Manchuria. The derived haplotypes within the main trees in Figures S1-S6 (Additional files 2, 3, 4, 5, 6 and 7) allow for inferences pertaining to the genetic origin of the populations and their relationships with each other. The age estimates, coalescence time, and variance computed from the roots and younger nodes are given in Table 1.

**Table 1:** Age estimates for haplogroups Y1a, G1b, D4e5, D4m, and N9b and their major subhaplogroups

Haplogroup	N	ML age (95% CI), kya
Y1	116	15.47 (9.09; 22.90)
>Y1a	101	10.56 (6.35; 15.35)
> > Y1a1	10	6.48 (3.22; 10.26)
> > Y1a-16189!	89	7.51 (4.58; 10.79)
G1b	61	11.35 (6.27; 17.08)
>G1b1	13	6.96 (3.41; 10.82)
>G1b-16129!	48	9.09 (5.06; 13.32)
D4e5	12	11.76 (5.56; 18.52)
>D4e5a	5	4.90 (1.72; 8.60)
>D4e5b	7	6.96 (2.61; 12.09)
D4m	24	17.50 (9.28; 27.06)
>D4m2	21	8.52 (4.46; 13.56)
> > D4m2a	19	6.25 (3.29; 9.66)
>> > D4m2a1	5	2.65 (0.80; 4.80)
>D4m1	3	3.98 (1.14; 7.18)
N9b	44	18.31 (12.58; 24.79)
>N9b1	16	15.47 (9.09; 22.90)
>N9b4	18	10.56 (6.35; 15.35)
M7a2	17	15.60 (9.74; 22.56)
>M7a2a	16	14.04 (8.89; 19.49)
> > M7a2a3	10	6.48 (3.10; 10.21)
>> > M7a2a3a	8	3.11 (0.90; 5.57)

### Haplogroup Y1a

mtDNA haplogroup Y (a descendant of haplogroup N9) is proposed to indicate matrilineal genetic continuity between late Pleistocene hunter-gatherer groups and present-day populations in the Far East [22, 25–29]. Within Siberia, the majority of contemporary Y1 carriers cluster into Y1a marked by the coding change m.7933A > G (aged ~ 10.6 kya), whereas Y1b and Y1c are confined to continental China, Japan, and Korea (Additional file 2: Figure S1). Accordingly, two offshoots arose from the Y1a founding haplotype for this haplogroup. On one side, the newly refined Y1a1 haplotype defined by m.12732 T > C is well represented in Tungusic-speaking groups (e.g., Evenki, Udegey), while the other side harbors a back mutation at np m.16189 T > C relative to the Reconstructed Sapiens Reference Sequence (RSRS). Sequence diversification within the Y1a-m.16189 T > C! haplogroup is characteristic (at most) of the Nivkhi from Sakhalin. The updated network analysis includes 10 Y1a2 sequences defined by m.12397A > G, of which 7 are new from the coastal Koryak.

### Haplogroup G1b

The newly reconstructed tree encompassing 61 mitogenomes (36 new and 25 published) illustrates the immediate split of G1b that created two offshoots. One is G1b1, defined by m.16207A > G, and the other is G1b-m.16129G > A! (Additional file 3: Figure S2). Whereas the G1b-m.16129G > A! cluster is exceptionally diverse, thus revealing the origins and relationships of the various cultures, G1b1 is much less prominent, being limited to a few sublineages. Notably, sequence data positive for m.16207A > G evidently shared an mtDNA haplotype of the G1b1 haplogroup among the Nivkhi, Ulchi, Coastal Koryak, and Itelmen [21–23].

### Haplogroup D4m2

The updated haplogroup D4m2 (D8 in [30]) is presented in Figure S3 (Additional file 4). The D4m2a sequences harbored by the Nivkhi are shown along with those from the Yukaghir, Evenki, Even, Tuvan, Buryat, Tubalar, and Tuvan from previous studies [22, 30–32]. Notably, the Nivkhi are the only bearers of D4m2a root haplotypes, while the Even, Evenki, and Yukaghir are grouped within the D4m2a1 subcluster.

### Haplogroups D4e5

We discovered D4e5b in 5 of 6 Oroki samples subjected to complete mtDNA sequencing (Additional file 5: Figure S4). Although an identical D4e5b sequence was previously identified in a sole Even individual (Nlk24) from a village on the Maya River, Okhotsk region (FJ858882), the HVS-I database indicates the major presence of D4e5b markers (m.16274G > A and m.16291C > T) among the Oroki from Sakhalin [33]. It appears that the most represented sublineage of D4e5b arose in the Amur basin 7.0 kya, whereas the age of the entire D4e5 haplogroup is 11.8 kya (Table 1), suggesting its ancestral homeland in interior Siberia and a subsequent split into two subclusters, D4e5a and D4e5b.

### Haplogroups N9b and M7a2

The Udegey group is found to consist of two mtDNA haplogroups: N9b and M7a2 (see Table 1 and Additional file 6: Figures S5 and Additional file 7: Figure S6). Aside from the Udegey originating in the villages of Gvasygi and Agzu in the Sikhote-Alin/Primorye region, we sampled the Udegey individuals who married into Ulchi and Nivkhi families dispersed along the reaches of the Lower Amur [22]. Haplogroup N9b is represented mainly by lineages of four major subhaplogroups: N9b1, N9b2, N9b3, and N9b4 [34–36]. We identified a novel N9b1 mitogenome (MH807371) in one individual from Primorye (Agzu), hence expanding the established geographical scope of the N9b1 haplogroup and disclosing a link between a component of the Udegey ancestry and the Hokkaido Jomon from Japan [36–38]. The second prevalent haplogroup is M7a2a3a, which was detected in 8/46 (17.4%) of the Udegey samples (Table 1, Additional file 7: Figure S6). The Udegey, as well as the Hokkaido Jomon, lack subhaplogroup M7a1, which is the predominant subhaplogroup in modern Japanese and Korean populations ([39] and ref. therein).

## Discussion

The phylogeographic dissection of matrilineal pools presented here revealed a wide range of distinct mtDNA lineages, some of which chronologically coincide with archeological phases of the Neolithic and could reveal matrilineal continuity between present-day populations and early Holocene forebears in the same region. It is not surprising to see considerable sharing of Y1a-m.16189 T > C! sublineages and haplotypes between the Nivkhi and Ulchi samples, given ethnographic evidence for mainland-Sakhalin interaction over the past several centuries. Notably, the Ulchi territory coincided with the meeting point of two trade routes, i.e., one via Lake Kizi and short portages leading to rivers flowing to the Tatar Strait and the other along the Amur to its estuary and to Sakhalin Island. Combined with their social position as silk trade middlemen officially sanctioned by the Qing administration, this was without a doubt a major factor leading to the formation of the Ulchi as a separate ethnic identity [3].

New complete sequences have refined the ancestral G1b type and hence implied genetic continuity between the Lower Amur and Kamchatka. The Lower Amur might have functioned as an incubator and ancestral homeland of the G1b root in the early Holocene before the split and subsequent spread of G1b-m.16129G > A! into higher latitudes. This conjecture is supported by the recent detection of ancient G1b in Duvanni Yar (Kolyma1), dated to ~ 9.8 kya, as well as at the Ol’skaya site, dated to ~ 3.0 kya, from the Magadan area, Chukotka [40]. Interestingly, mtDNA data from previously published studies on Russian old settlers in the Kolyma-Indigirka-Anadyr region, which relates to Yukaghir history, reveal high frequencies of G1b-m.16129G > A! [22, 30, 41, 42].

Despite the fact that the D4m2 haplogroup is scattered throughout a vast territory stretching over northern China and Mongolia, the Russian Far East and North Siberia, its frequency and diversity across the entire area are low, with the Lower Amur and Primorye being exceptions. Important caveats include single nucleotide polymorphism (SNP) sequences related to D4m in Neolithic remains (5726–5622 cal BC) from the Devil’s Gate cave sites in Primorye [40, 43], thus indicating long-standing genetic succession in this region during the Holocene.

From the phylogenetic network (Additional file 6: Figures S5 and Additional file 7: Figure S6), it is possible to infer that the Udegey represent admixture of southern Siberian populations and the northern Jomon people. Interestingly, Wang et al., 2020 [44] reported data on Jomon hunter-gatherers from Japan who harbored one of the earliest splitting branches of the East Eurasian variation and showed an affinity among the Jomon, the Amur River Basin, ancient Taiwan, and Austronesian speakers, as expected for their ancestries if they all had contributions resulting from late Pleistocene coastal route migration to East Asia. Taken together, these mtDNA findings demonstrate strong genetic overlap between the mitogenome pool of modern autochthonous populations and ancient groups of the Russian Far East.

## Conclusion

Here, we extended the survey of major mitochondrial lineages dispersed across the Russian Far East. Several components may be delineated in this regard. The first component traces back to East Eurasian hunter-gatherers and represents lineages belonging to subdivisions of haplogroups N9b and M7a2. The second is well represented by Y1a and G1b and points to the Lower Amur as the ancestral homeland for this and other haplogroups. The third comprises D4e5, which establishes an association between the Oroki and interior eastern Eurasian populations. Last, rare D4m2a mtDNA exhibited by modern Siberians may have roots in Primorye, at the eastern edge of the continent, rather than a South-Central Siberian source. The data obtained have provided new insights into long-standing questions pertinent to the nature and timing of human colonization of Northeast Asia.

## Methods

### mtDNA genome data analysis

Genomic DNA was extracted from blood buffy coats using standard procedures. The complete sequencing procedure for modern samples entailed PCR amplification of 2 overlapping mtDNA templates that were sequenced with an Illumina HiSeq 2000. Short reads were mapped using bwa version 0.6.1 [45]. All mtDNA genome consensus sequences were called using Unipro UGENE version 1.21 software [46]. We embedded modern and ancient mtDNA genomes in the existing mitochondrial tree of PhyloTree using mtPhyl version 5.003 [47]. mtPhyl follows the hierarchical structure of the tree up to the most derived SNP shared with an existing sequence haplotype. Variants were scored relative to the RSRS [48], with common indels and mutation hotspots at nucleotide positions m.309.1C(C) and m.315.1C and AC indels at m.515-m.522, m.16182A > C, m.16183A > C, m.16194A > C, and m.16519 T > N being excluded [49]. The haplogroup affiliations reported in this analysis correspond to the current nomenclature of mtDNA in agreement with the latest release of PhyloTree-Build 17 [49].

BEAST version 1.10.1 was used to calculate maximum likelihood (ML) estimates for complete Y1a, G1b, D4m, D4e5, M7a2 and N9b sequences with a log-normal relaxed molecular clock (uncorrelated), using ancient samples as calibration tips [50, 51]. Mutational distances were converted into years using the substitution rate for the entire molecule of 2.67 × 10^− 8^ substitutions per site per year [51]. The general time-reversible sequence substitution model with a fixed fraction of invariable sites and gamma-distributed rates (GTR + I + G) was used because this model had the best fit to our data according to jModelTest version 2.1.4 [52]. Using the Markov chain Monte Carlo (MCMC) approach, 50,000,000 iterations were performed, with samples taken every 1000 steps. The initial 10% of iterations were considered burn-in and discarded. Inspection of posterior samples ensured sufficient sampling and was used to check for convergence to the stationary distribution.

## Supplementary information

**Additional file 1 **: **Table S1.** List of mitogenome sequences included in Figures S1-S6.

**Additional file 2 **: **Figure S1.** Phylogenetic tree of haplogroup Y1a.

**Additional file 3 **: **Figure S2.** Phylogenetic tree of haplogroup G1b.

**Additional file 4 **: **Figure S3.** Phylogenetic tree of haplogroup D4m.

**Additional file 5 **: **Figure S4.** Phylogenetic tree of haplogroup D4e5.

**Additional file 6 **: **Figure S5.** Phylogenetic tree of haplogroup N9b.

**Additional file 7 **: **Figure S6.** Phylogenetic tree of haplogroup M7a2.

## Data Availability

The sequence data for the new mitogenomes (*n* = 104) that support the findings of this study have been deposited in the National Center for Biotechnology Information (GenBank; http://www.ncbi.nlm.nih.gov/Genbank/) under accession numbers MG660751-MG660801 and MH807322-MH807374.

## Abbreviations

mtDNA: Mitochondrial DNA
Mitogenome: Mitochondrial genome
Cal BC: Radiocarbon-calibrated date Before Christ
Kya: Thousand years ago
Hg: Haplogroup
ML: Maximum likelihood
HVS-I: Hypervariable Segment I
RSRS: Reconstructed Sapiens Reference Sequence
PCR: Polymerase chain reaction
MCMC: Markov chain Monte Carlo
