# Supplementary material for: Genetic legacy of cultures indigenous to the Northeast Asian coast in mitochondrial genomes of nearly extinct maritime tribes
Source: BMC Evol Biol. 2020 Jul 13;20:83. doi: 10.1186/s12862-020-01652-1 (PMC7359603; doi:10.1186/s12862-020-01652-1)

Figure S1. Maximum parsimony phylogenetic tree of haplogroup Y1a: the sequences incurred are from Table S1. The sequences in red are generated through the course of this study. When two or more identical sequences belong to the same branch, their number is given in brackets. We use PhyloTree annotation: mutations are transitions unless a specific base change was specified; position number followed by a dot (.) precedes the insertion; back mutation and double back mutation are indicated with an exclamation (!) and double exclamation (!!), respectively; mutations between brackets () are recurrent/unstable within the respective clade, or are uncertain yet.

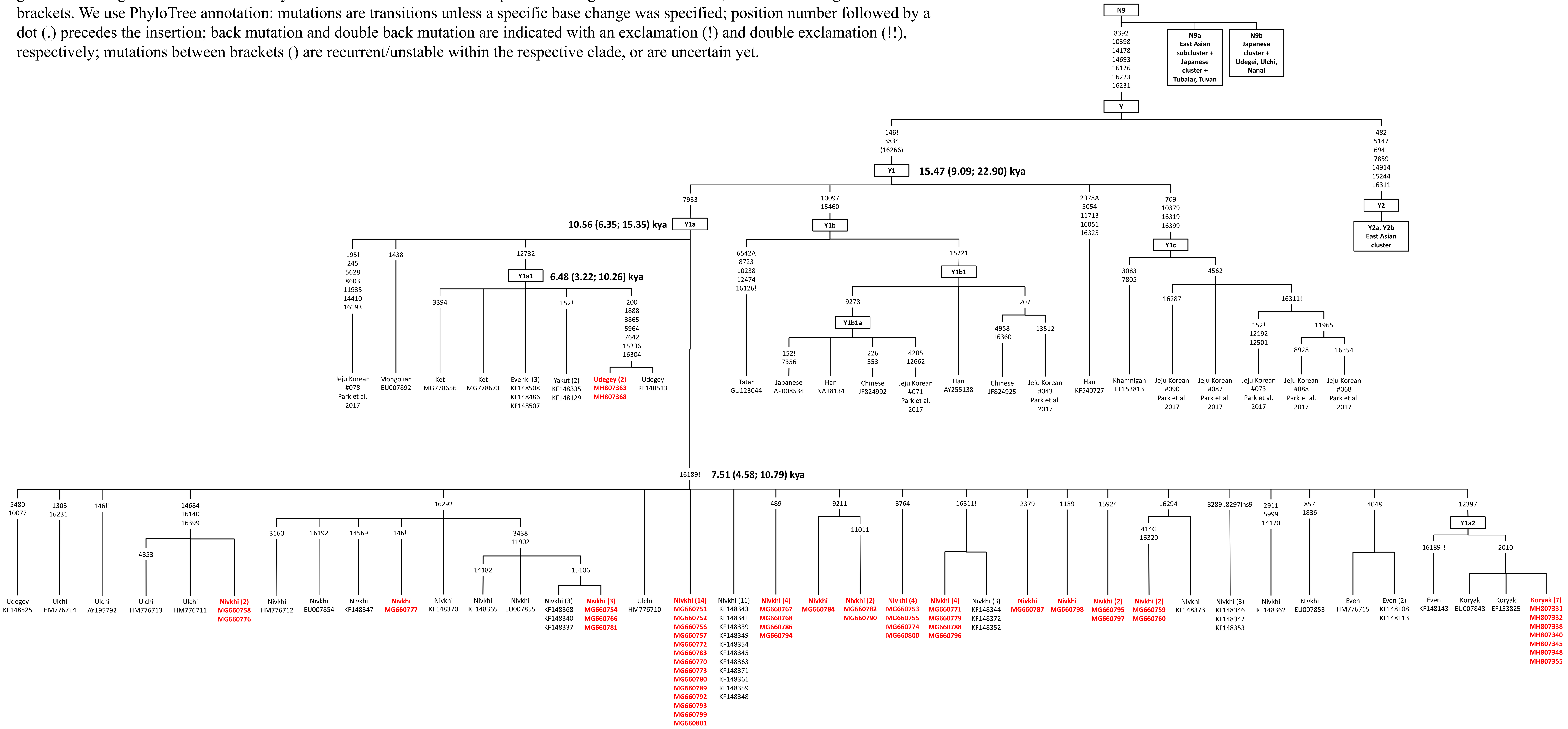

Supplement: Supplementary file 2 — Additional file 2 : Figure S1. Phylogenetic tree of haplogroup Y1a. [file 12862_2020_1652_MOESM2_ESM.pdf]
