# Supplementary material for: Genetic legacy of cultures indigenous to the Northeast Asian coast in mitochondrial genomes of nearly extinct maritime tribes
Source: BMC Evol Biol. 2020 Jul 13;20:83. doi: 10.1186/s12862-020-01652-1 (PMC7359603; doi:10.1186/s12862-020-01652-1)

Figure S3. Maximum parsimony phylogenetic tree of haplogroup D4m: the sequences incurred are from Table S1. The sequences in grey are ancient which gleaned from Sikora et al., 2019; dashed lines for ancient samples indicate that the sequence is not complete. When two or more identical sequences belong to the same branch, their number is given in brackets. We use PhyloTree annotation: mutations are transitions unless a specific base change was specified; position number followed by a dot (.) precedes the insertion; back mutation is indicated with an exclamation (!).

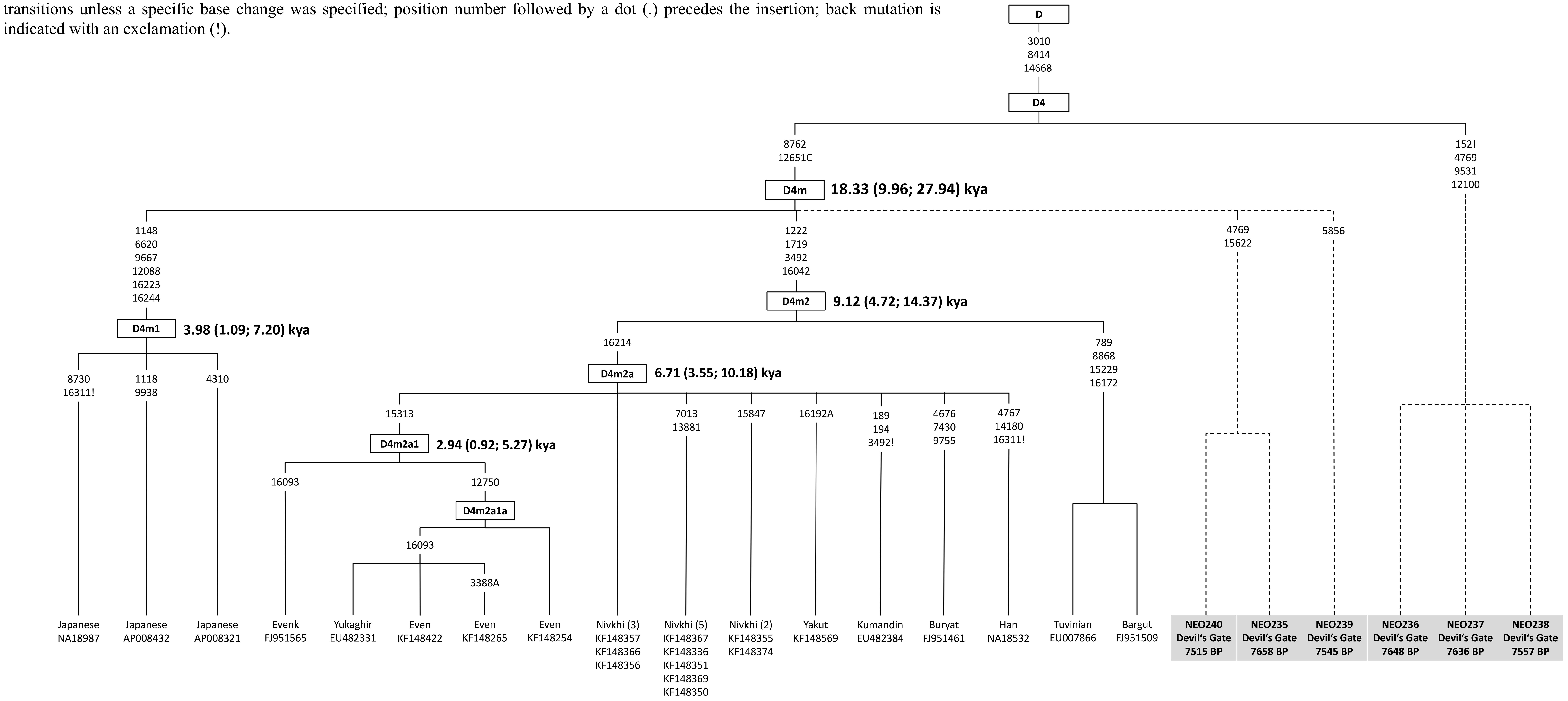

Supplement: Supplementary file 4 — Additional file 4 : Figure S3. Phylogenetic tree of haplogroup D4m. [file 12862_2020_1652_MOESM4_ESM.pdf]
