# Supplementary material for: Genetic legacy of cultures indigenous to the Northeast Asian coast in mitochondrial genomes of nearly extinct maritime tribes
Source: BMC Evol Biol. 2020 Jul 13;20:83. doi: 10.1186/s12862-020-01652-1 (PMC7359603; doi:10.1186/s12862-020-01652-1)

Figure S4. Maximum parsimony phylogenetic tree of haplogroup D4e5: the sequences incurred are from Table S1. The sequences in red are generated through the course of this study. When two or more identical sequences belong to the same branch, their number is given in brackets. We use PhyloTree annotation: mutations are transitions unless a specific base change was specified; position number followed by a dot (.) precedes the insertion; back mutation is indicated with an exclamation (!).

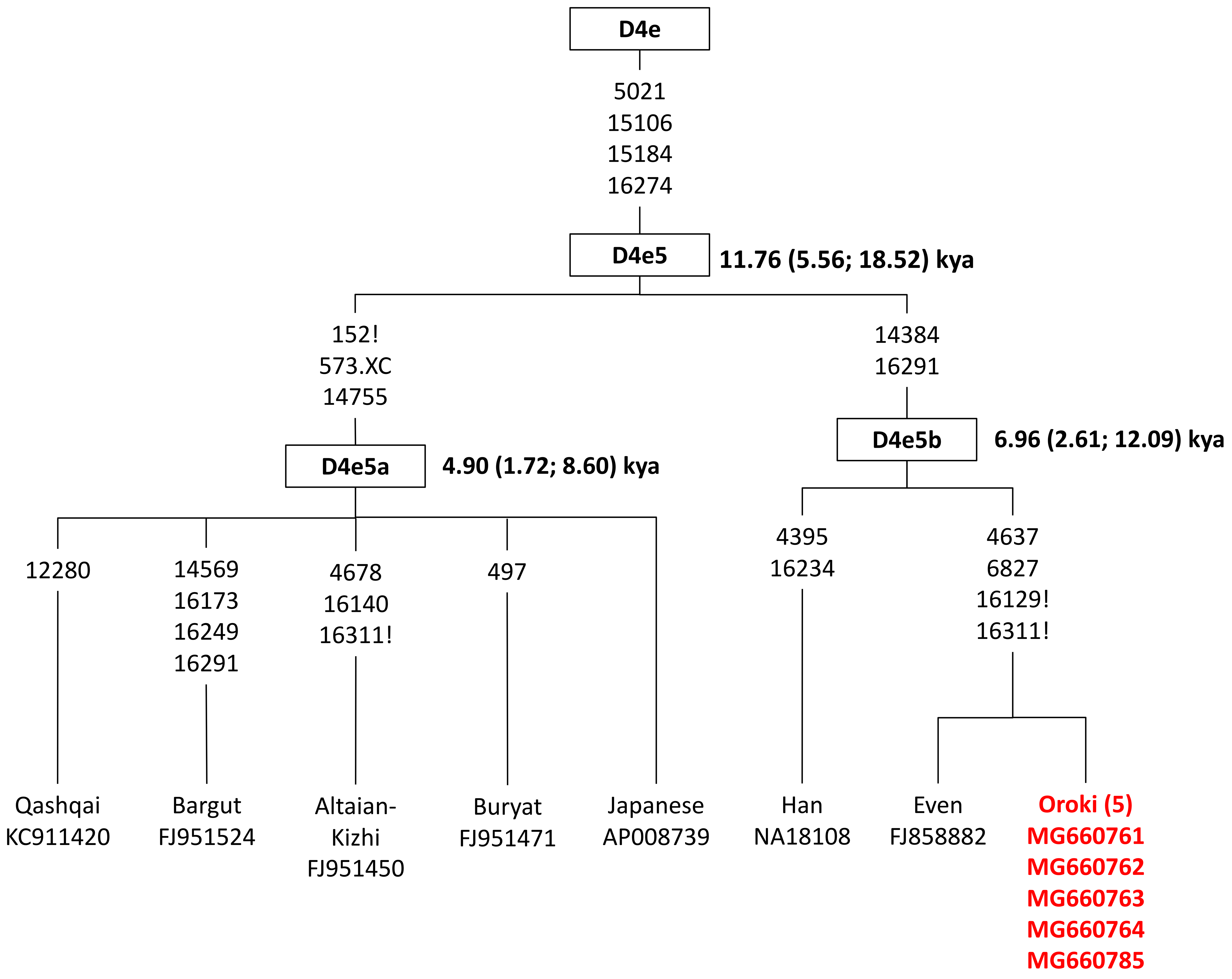

Supplement: Supplementary file 5 — Additional file 5 : Figure S4. Phylogenetic tree of haplogroup D4e5. [file 12862_2020_1652_MOESM5_ESM.pdf]
